# Transcranial direct current stimulation to enhance athletic performance outcome in experienced bodybuilders

**DOI:** 10.1371/journal.pone.0220363

**Published:** 2019-08-01

**Authors:** Ali-Mohammad Kamali, Zahra Kheradmand Saadi, Seyedeh-Saeedeh Yahyavi, Asadollah Zarifkar, Hadi Aligholi, Mohammad Nami

**Affiliations:** 1 Department of Neuroscience, School of Advanced Medical Sciences and Technologies, Shiraz University of Medical Sciences, Shiraz, Iran; 2 DANA Brain Health Institute, Iranian Neuroscience Society-Fars Branch, Shiraz, Iran; 3 Neuroscience Laboratory, NSL (Brain, Cognition and Behavior), Department of Neuroscience, School of Advanced Medical Sciences and Technologies, Shiraz University of Medical Sciences, Shiraz, Iran; 4 Student research committee, Shiraz University of Medical Sciences, Shiraz, Iran; 5 Department of Foreign Languages and Literature, Shiraz University, Shiraz, Iran; 6 Department of Physiology, School of Medicine Shiraz University of Medical Sciences Shiraz Iran; 7 Academy of Health, Senses Cultural Foundation, Sacramento, California, United States of America; University of Waterloo, CANADA

## Abstract

Transcranial direct current stimulation (tDCS) is currently under investigation as a promising technique for enhancement of athletic performance through modulating cortical excitability. Through consecutive randomization, 12 experienced bodybuilders were randomly assigned to two arms receiving either sham or real tDCS over the primary motor cortex (leg area) and left temporal cortex (T3) for 13 minutes in the first session. After 72 hours, both groups received the inverse stimulation. After the brain stimulation, cerebral hemodynamic response (using frontopolar hemoencephalography) was examined upon taking three computer-based cognitive tasks i.e. reasoning, memory and verbal ability using the Cambridge Brain Science-Cognitive Platform. Subsequently, the bodybuilders performed knee extension exercise while performance indicators including one-repetition maximum (1RM), muscular endurance (SEI), heart rate (ECG), motivation (VAS), surface electromyography over quadriceps femoris muscle (sEMG) and perceived exertion (RPE) were evaluated. The real tDCS vs. sham group showed decreased RPE and HR mean scores by 14.2% and 4.9%, respectively. Regarding muscular strength, endurance, and electrical activity, the 1RM, SEI, and sEMG factors improved by 4.4%, 16.9%, and % 5.8, respectively. Meanwhile, compared to sham, real tDCS did not affect the athletes’ motivation. Incidentally, it turned out that subjects who underwent T3 anodal stimulation outperformed in memory (p = 0.02) and verbal functions (0.02) as well as their corresponding frontopolar hemodynamic response [(memory HEG (p = 0.001) and verbal HEG (p = 0.003)]. Our findings suggest that simultaneous tDCS-induced excitation over the M1 leg area and left temporal area may potentially improve the overall athletic performance in experienced bodybuilders (Trial registration: IRCT20181104041543N1, Registered on 4 Nov. 2018, retrospectively registered).

## Introduction

In competitive sports, three principles including faster, higher, and stronger hold significant importance in not only professional but also amateur athletes. Over recent years, there has been an increasing interest in brain stimulation and neuromodulation to cross-link neuroscience and athlete’s performance [1]. One of the brain stimulation methods is transcranial direct current simulation (tDCS) which results in brain excitability changes through a weak direct current. In 2013, Davis coined the word ‘neurodoping’ which is representative of using advanced techniques for mental and physical enhancement of athletes [2]. The compelling idea of incorporating neuroscience in sport as well as he relationship between industry and science has led to the knowledge-based products for improving professional athletes’ performance (www.haloneuro.com) [2]. In fact, factors such as motor learning, muscular strength, fatigue or even processing speed for specific motor skills may gain through non-invasive brain stimulation methods [3].

tDCS transmits a weak current (1 to 2 mA) through surface electrodes over scalp typically for the duration 5 to 20 minutes. This electrical current is transferred to brain tissue and affects the excitability and neuronal activity of the brain. In other words, tDCS changes the action potential threshold in neurons [4]. Anodal tDCS leads to depolarization in resting membrane potential and axons of target neurons resulting in an increased neuronal firing rate and cortical excitability [5]. On the other hand, cathodal tDCS leads to decreased excitability through hyperpolarization [5]. Studies have indicated that the effect of at least 10 minutes brain stimulation would last an hour after the intervention [5]. It is presumed that a similar plasticity trend exists in glutamatergic neurons [5]. This tDCS-induced modulation is evident in fMRI studies where anodal and cathodal stimulation increases and decreases blood oxygen level-dependent (BOLD) response in targeted areas, respectively [6].

An earlier report indicated the positive effects of tDCS over the right motor cortex of healthy subjects in increasing isometric endurance of left elbow, decreasing muscle fatigue, and improving motivation and muscle synergy [7]. Similarl, another study showed the effectiveness of anodal tDCS over the motor cortex for improving muscular endurance, decreasing fatigue, and enhancing athletes’ performance [8].

Moreover, anodal tCDS over the temporal cortex (TC) has been found to modulate the activity of autonomic nervous system (ANS) and improve peak power output of trained cyclists by reducing their perceived exertion (PE) and heart rate (HR) [9]. TC has been associated with ANS- autonomic dysfunction during or after seizures and may result in cardiac and pulmonary changes [10]. In another study, tDCS over the left temporal lobe, increased HR variability which was indicative of improving parasympathetic modulation of HR [11]. It should be noted that higher vagal modulation enhances the autonomic cardiac function where physical fitness is attributed to cardiac vagal function during exercise [12]. Compared to non-athletes, athletes have higher vagal modulation and their HR increase more slowly in a specific motor task [12]. In case that tDCS can change brain areas associated with ANS and increase vagal modulation, it can improve athletes’ performance during training. Vagal modulation changes can be assessed by HR changes before and after tDCS [9].

With respect to motor functions, an investigation showed that anodal tDCS over M1 improved the cycling performance and increased time to exhaustion; however, no significant changes were reported for PE and HR factors [13].

In addition to the effectiveness of tDCS for muscular fatigue, a recent research demonstrated the positive effect of dlPFC stimulation on implicit motor learning [14]. The study which was done on 27 healthy individuals showed that cathodal stimulation of dlPFC compared to sham tDCS improved golf putting performance suggestive of enhanced implicit motor learning. Nevertheless, the participants’ verbal working memory was impaired.

Previously, modulation of motor cortex through the application of tDCS had been studied [15] whereby anodal stimulation of the M1, premotor, or prefrontal cortices during a reduction time task indicated the active role of M1 in implicit motor learning, while the stimulation of other areas showed no specific effect on the same. In line with this study, another research examined the effects of tDCS over M1 on motor skill acquisition and its long-term retention. The findings showed that anodal tDCS group developed a better skill acquisition trend compared to the sham group [16].

Given the importance of motivation in performance and physiological response of the athletes, the efficacy of tDCS on motivational level of athletes has been addressed in some reports [17, 18].

In one of our research-team’s reports, anodal and cathodal tDCS over the left prefronral and ipsilateral cerebellar cortices, respectively, in professional pistol shooters could improve shooting task scores [19]. However, this emerging field requires more research to define the effectiveness of tDCS on athletes in various sport field as well as the optimized protocols including stimulation duration, electrode montage and stimulation amplitude for tDCS application in sport.

Furthermore, most of the studies done till now have examined a small muscle mass such as biceps brachialis. Thus, examining a rather big section of muscles can be of greater significance. So far, there is no study addressing the effects of tDCS on weight lifting exercises.

Nevertheless, some studies have shown that tDCS cannot enhance motor functions. For instance, one report indicated that stimulation of the right motor cortex (2mA) did not exert any significant effect on the neuromuscular fatigue [20]. Another study showed that tDCS did not improve muscular performance in an isometric exercise. The authors concluded that since the muscle has already been reaching its maximal strength capacity, the intervention could not further enhance the muscular strength [21]. Nevertheless, the majority of sports-related studies into the motor cortex have shown the positive significant effects of tDCS [22].

That said, the present investigation was designed to examine the effects of anodal tDCS over M1 leg area and TC on muscular power, short-term muscular endurance, subjective fatigue perception, HR, cognitive functions, frontopolar hemodynamic response, and motivation towards the lifting task. The study primarily hypothesized that our intervention vs. sham condition would lead to an enhanced short-term muscular endurance, decreased subjective fatigue perception, decreased post exercise HR, increased frontopolar hemodynamic response, and motivation while cognitive functions are sustained or even improved.

## Method

### Ethical approval and consent to participate

This was a factorial single-armed randomized trial in which subjects were assigned to sham or true tDCS intervention through simple randomization in 1:1 ratio. Approval for this study was obtained from the Shiraz University of Medical Sciences (SUMS) (No. 1396-01-74-14298). Since the Iranian Registry of Clinical Trials (IRCT) registration process is time-consuming, in some cases the university’s review board allows trial commencement based on the permission granted by the institution. The first participant’s recruitment was then done based on the ethical board approval (IR.SUMS.REC.1396.147 granted on 4^th^ April 2017) and the permission granted by the institutional review board committee at SUMS (1396-01-74-14298). The work was also registered by IRCT under the code IRCT20181104041543N1 (granted on 23^rd^ Dec 2018) and the registration timing was retrospective.

Recruitment was done during the period of 4^th^ April 2017-22^nd^ October 2018.

The authors confirm that all ongoing and related trials for this intervention are registered.

### Participants

Informed consent: The entire process including its rationale and objective, the participants' role and safety consideration was explained to each candidate in plain language. The participants were then asked to sign a written informed consent indicating that their data would remain confidential and they may resign from the process on their discretion whenever during the project. The consent was made in two identical copies of which the participants could retain one.

Sampling method, case selection: Case selection followed a cluster random sampling method. The bodybuilding sport clubs were alphabetically sorted and randomly approached within various district of the town. Candidates (experienced bodybuilders) who had at least 2 years of consistent bodybuilding exercise (minimum of three sessions per week lifting workouts) were sequentially selected and debriefed about the project to get possibly enrolled.

After random selection of 6 bodybuilding gyms across the city of Shiraz and posting announcement in the gyms, 12 experienced bodybuilders were randomly chosen from those who volunteered to participate in the study.

With respect to sample size calculation, we referred to the earlier related reports [13, 19, 23–25] in which the sample size ranged from 8 To 16 The Kelsey and Fleiss sample size calculation formula [26] was used (power 80 and α = 0.05) whereby the minimum justifiable number of 20 participants were decided to get enrolled. Based on earlier reports in the field of sport, the specialized population namely bodybuilders limited the sample size of the study. Those who enrolled in the study were males aging 18 to 40 with weight between 60 to 120 kg who were regularly training weightlifting exercise (at least 3 times a week) not on doping drugs for at least 3 months prior to enrollment. The participants were assured not to have psychological or neurological disorders or a history of alcohol or drug use. In addition, the volunteers were instructed to refrain from vigorous activities and the ingestion of beverages containing caffeine and alcohol or of using tobacco for 24 h prior to each test.

Since subjects' performance was compared to their own sham-stimulation status, the use of ordinary food supplements was excluded from our "red-flag" checklist. Meanwhile the use of medicaments and specific supplements (within three months prior to enrollment) which were indicated in the official list of the World Anti-doping Agency (2018) was considered as an exclusion criterion.

The above three-month time window was decided since majority of the doping listed agents can hardly be traced in regular anti-doping test after such a period.

After all, the study design (within group self-comparison upon sham and true brain stimulation) could potentially minimize the biasing effect.

### Experimental design

**Fig 1:**
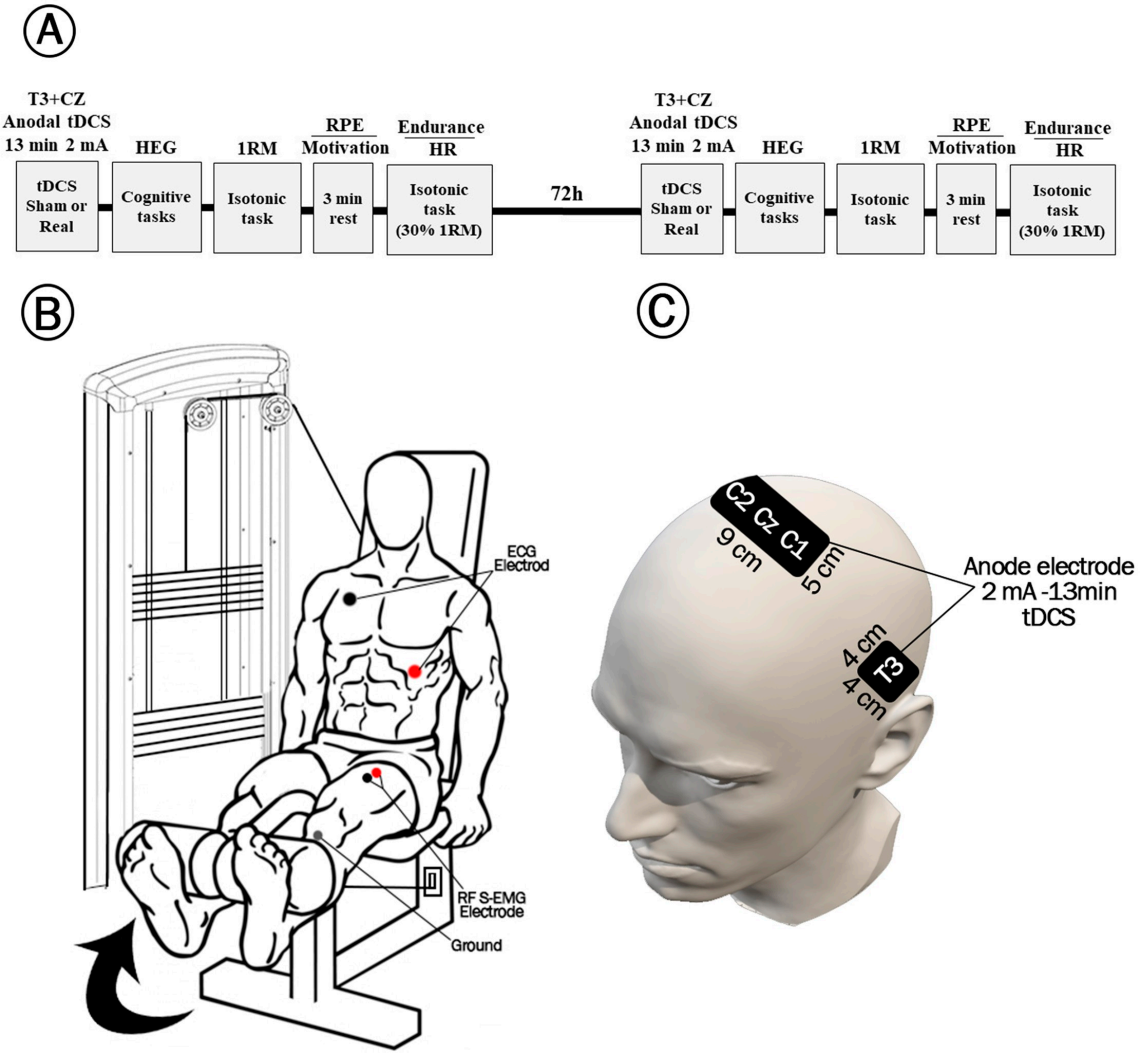
Study protocol, leg extension exercise involving quadriceps muscles, and the tDCS montages used for brain stimulation. **(A)** Participants were randomly assigned to either sham or real tDCS at 2mA for 13 minutes over the first session. Then, they performed 3 tasks including reasoning, memory and verbal from CBS-cognitive platform (see Methods section) with the intervals of 3 minutes’ rest. CBS-CP and the HEG data were concurrently recorded while subjects carried out the tasks. Later, they performed the leg extension exercise and their 1RM were recorded. With the interval of 3 minutes, the participants’ perceived exertion was examined and then, they performed the leg extension with 30% of their 1RM and their endurance level was recorded. Meanwhile, their heart rate during the exercise was also recorded. After 72 hours, the real group received sham tDCS whereas sham group received real tDCS for 13 minutes and they performed the rest of the tasks similar to the first session. **(B)** The examinees were supposed to choose a weight, bend the legs at a 90-degree angle and after extending the legs, move them back to the primary position. To calculate the 1RM, the athletes were asked to perform the exercise for at least 6 to 12 times with the maximum weight. For the SEI, they were required to bear 30% of their 1RM weight and perform the exercise as many times as they could. The sEMG sensors were attached to the midpoint of anterior superior iliac spine and patella through chest leads with the ground on patella and the sEMG data were recorded during the 1RM exercise. The ECG sensors were attached to the upper right portion of chest below collarbone and below left breast over lower rib-cage to obtain recording during the endurance exercise. **(C)** A 2 mA anodal tDCS pad electrode was placed over the left temporal cortex (T3) and anodal tDCS over the Cz covering C1 and C2 (M1 leg area) for a course of 13 minutes. The size of the electrodes is depicted in the Fig. The cathode electrodes for M1 and T3 were placed over the right and left shoulders, respectively.

Through a double-blind, counterbalanced design, a clinical neuroscientist who was blind to session assignment used a random digit table to sequentially allocate subject to interventions. Data were obtained over two sessions with the interval of 72 hours (Fig 1A). A researcher who was blinded to data collection process randomly assigned the participants to sham and real tDCS arms using consecutive randomization. Subjects were randomly submitted to 2 mA sham or real tDCS over M1 and TC for 13 minutes in the first session. After 72 hours, the group who received sham first, received real tDCS and the first real tDCS group received sham in the second session. Since the participants were under maximum physical pressure during each testing session, a proper interval was required between the two sessions. The 72h intermission was therefore considered to avoid the confounding effect of muscular fatigue. Moreover, a day before the study (for both sessions), the participants were contacted via phone and reminded to get some quality sleep and maintained their routine diet. In addition, they were asked to avoid coffee, alcohol, and other exclusion criteria on the day of the study. In addition, the study design (random true or sham stimulation in the first or second session) could reduce the effect of inter-day variability in performance over the two different sessions. The Visual Analogue Scale was used to measure the participants” fatigue on the study day to exclude those with subjectively reported excessive fatigue.

It is shown that the effects of 13 minute 2mA stimulation on cortical excitability would fade after 150 minutes [27]. Following the brain stimulation, to examine the participants’ cognitive performance, the bodybuilders were required to perform 3 tasks from the computerized Cambridge Brain Sciences-Cognitive Platform (CBS-CP) with the intervals of 3 minutes between each task. Meanwhile, their prefrontal hemodynamic response was evaluated using the hemoencephalography (HEG) setup upon performing the mentioned tasks. The participants were then asked to warm up and perform the knee extension exercise for at least 6 to 12 times with the maximum weight that they could bear with the Knee Extension Machine in order to obtain their 1RM (Fig 1B). This exercise is considered as one of the basic moves in weightlifting practice targeting the quadriceps femoris muscle. It should be noted that only the practices in which the legs could bend at a 90-degree angle were recorded as correct moves.

After 3 minutes rest, in order to assess the participants’ exertion rate upon knee extension exercise for obtaining 1RM, the bodybuilders were asked to fill the hr questionnaire [28]. Earlier reports have postulated a high correlation between RPE and blood as well as muscle lactates which are biochemical indicators of heart and muscle exertion [28]. Furthermore, to assess the participants’ motivation in continuing the exercises, a Visual Analogue Scale (VAS) was used. After that, participants were asked to choose a weight equaling 30% of their 1RM and perform the knee extension exercise as many times as they could. This time, multiplication of the weight by the number of successive exercise was considered as the Short-term Endurance Index (SEI). Moreover, the participants’ HR during exercise performance was recorded as an indicator of autonomic response.

Taken together, the study randomized the subjects into sham and real tDCS then switched and compared results between stimulations. The difference in variable scores following true- vs. sham-tDCS (through paired *t*-test) was attributed to the potential effect of the intervention.

### Transcranial direct current stimulation (tDCS)

In this study, a two channel tDCS device (Neurostim-2, Medina Teb) was used to transfer a 2mA electrical current for 13 minutes with ramping up and down of 30 seconds. In one channel, the anode electrode was placed over the Cz (35 Cm^2^) overlying C1 and C2 (M1 leg area) responsible for leg movement and the cathode (16 Cm^2^) was placed over the right shoulder. For the second channel, the anode (16 Cm^2^) was placed over the TC (T3) and the cathode electrode (16 Cm^2^) was placed over the left shoulder. The electrodes were placed based on the international 10–20 EEG electrode placement system and the saline-soaked sponges (NaCl 150 mM) were used under the electrodes over the scalp. To induce a sense of stimulation in the sham session, an electrical current was delivered for 30 seconds and then, the current was switched off; however, the count-down indicator and the indicator light on the device screen were on throughout the session. In the real tDCS session, the electrical current was delivered for 13 minutes.

According to some studies, the stimulation of motor cortex for 10 minutes does not have a significant effect on muscular performance. Nitsche et al. [27] showed that the effects of a tDCS session (2mA, 13min) continued to remain for 150 minutes. In addition, some other studies into cyclists indicated the effectiveness of stimulation (13min) in enhancing their athletic performance [13]. As a result, we considered a stimulation for 13 minutes as an optimal stimulation duration already examined. It is worth noting that the length of stimulation was 20 minutes in the majority of studies. We considered a shorter length of stimulation as it could be more convenient before sport competitions.

With regard to the stimulation sessions, subjects were briefed about the possible fine tingling sensation they might feel during the stimulation. They were also reassured about the safety profile of the process. During the tDCS session (13 min), the participants were instructed to sit still comfortably on a chair and do nothing, keeping their eyes open.

At the end of each stimulation sessions, the participants were asked about their feeling regarding the sham versus real tDCS. Since the participants were stimulated for 30 minutes in the sham session with 30 second ramp—up, they could not accurately distinguish between the sensation over the real and sham sessions.

The tDCS montage used for brain stimulation is depicted in Fig 1C.

### Instruments, measurements and metrics

The instruments and materials used upon data collection.

**Visual Analogue Scale (VAS):** used as a continuous single-item fatigue scale ranging from 0 (no fatigue) to 10 (severe fatigue). This scale evaluated the participants’ motivation in performing the tasks and continuing the exercises.

**Heart Rate (HR) recording:** the HR was recorded by a NeXus-4 (MindMedia, Netherlands) Biofeedback setup. The electrocardiography (ECG) sensors were attached to the participants through chest leads and the HR data were recorded while the participants were working out the knee extension endurance exercise. The average HR of each two knee extension exercises was calculated for further analysis.

**Rated Perceived Exertion (RPE):** This is a 6 (no exertion) to 20 (maximal exertion) scale developed by Borg et al. [28] to assess the body PE during exercise. The RPE was shown to the participants and they were instructed how to report their PE. The reliability index of this scale reported by Borg et al. was robust (r = 0.92). Our study employed the validated Persian version for the same purpose.

**One-Repetition Maximum (1RM) scale:** muscle strength is the capacity of a muscle to exert force. 1RM is regarded as one-repetition maximum used to determine maximum strength calculated through 1RM = *w* ($1+\frac{r}{30}$), considering *r* >1 [29], where *r* is the number of repetitions performed and *w* is the amount of weight participants could lift by their knee extension.

**Short-term Endurance Index (SEI):** To assess this factor, the participants were asked to choose 30% of their 1RM and perform as many as the knee extension exercise they can. The SEI was calculated by multiplication of the amount of weight (30% of their 1RM) by the number of successive exercise.

**The Cambridge Brain Science Cognitive Platform (CBS-CP):** Cognitive performance is a crucial substrate of athletic function in many instances [30, 31]. In order to distinguish the positive or negative impact of our brain stimulation protocol on cognitive performance of the participant’s cognitive assessment was pursued. To do so, a media-rich computerized online platform addressing three higher-order cognitive components of reasoning, memory and verbal ability [32] was used. From each component, a test was chosen to see the effects of tDCS on different aspects of cognitive performance. The ‘Rotation, Monkey Ladder and Digit Span’ tasks were chosen from reasoning, memory, and verbal domains, respectively.

**Prefrontal hemodynamic response:** the assessment was used to identify local intracranial hemodynamic changes in prefrontal cortex (PFC) using a Hemoencephalography (HEG) device (a peanut near infra-red HEG kit, BIOCOMP Research Institute, Los Angeles, CA). Thereby, the optical density in left frontopolar (FP1) area was recorded during completion of the three aforementioned CBS-CP tasks after either sham or real tDCS intervention.

**Surface Electromyography (sEMG):** A Nexus Biofeedback setup was used to record sEMG from the rectus femoris muscle, a factor representing the neuromuscular dynamics. Due to limitations in the device channels, we had to stimulate only one muscle and record the heart rate at the same time with other channel of the polygraphy device. The rectus femoris muscle is one of the most important muscles involved in knee extension. Since the motor cortex of the leg was stimulated through tDCS, the contraction of this muscle was considered as a sample resembling the activity and contraction of the rest of muscles within quadriceps. However, the selection of this muscle does not indicate its superiority over other quadriceps femoris muscles.

Before placing the electrodes, the site was shaved, disinfected by alcohol, and given time to dry out completely. Employing Surface EMG for Non-Invasive Assessment of Muscles (SENIAM) guidelines [33], the sensors were attached to the midpoint of anterior superior iliac spine and patella through chest leads and the sEMG data were recorded during the 1RM exercise. The sEMG recordings were pre-processed and denoised to eliminate peaked sharp artifacts. EMG sampling rate was 1,024 per second. A band pass filter from 100 Hz to 500 Hz was applied during online recording. Raw EMG data were then recalculated through the root mean square (RMS) method to transform EMG signals into amplitudes. The resulting amplitudes were then subject to statistical analysis.

In addition to raw signals, Biotrace+ software (V2017A, Mind Media B.V., The Netherlands) provided root mean square data (epoch size: 1/8 s, 32 samples per second). Results are illustrated in Fig 4.

### Data analysis

Based on the normality of distribution, parametric and non-parametric statistical tests were employed. A series of paired sample *t*-tests were run to compare the differences between sham and real tDCS with regard to different factors including motivation, HR, RPE, 1RM, SEI, CBS-CP, and HEG data.

Wilcoxon signed-rank test was used to analyze data lacking normal distribution. The differences between the sham and real tDCS sessions were evaluated based on the Mean±SEM (Standard Error of Mean). The *p* values below 0.05 were considered as statistically significant. The SPSS statistical package (Version 22.0.0) was used for data analyses.

## Results

12 experienced bodybuilders were randomly chosen from those who volunteered to participate in the study.

The participants’ demographic data [Mean±SEM (standard error of mean)] included: age in years = 25.6±6; years of training in bodybuilding = 5.7±3.4 and years of formal education = 15±3.

**Fig 2:**
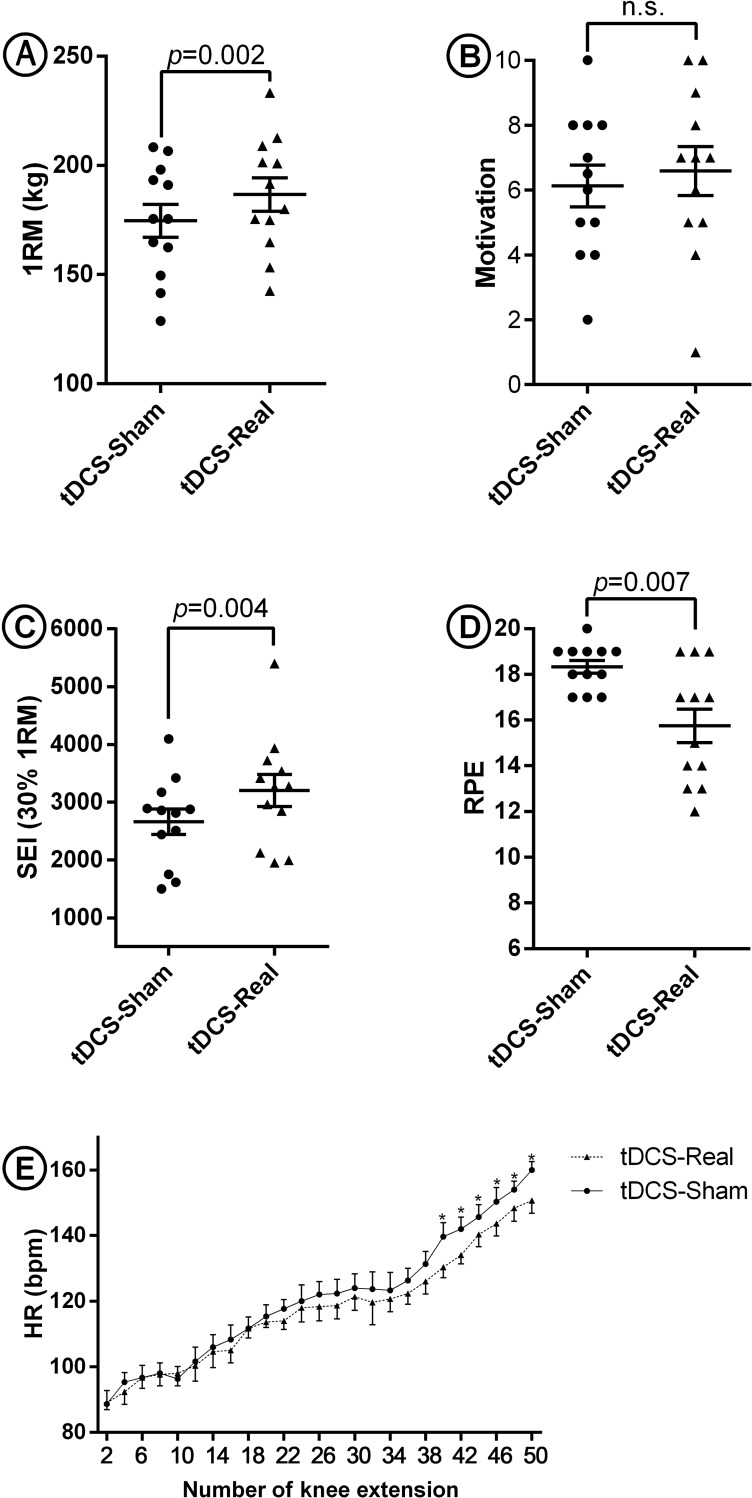
Dot plots representing the participants’ performance for the task outcomes including muscular strength (1RM), motivation, muscular endurance (SEI), perceived exertion (RPE), and HR. Panel a shows a significant difference between the 1RM of the athletes in true vs. sham tDCS sessions (p <0.05). The 1RM was calculated through 1RM = w ($1+\frac{r}{30}$), considering r >1 (48), where r is the number of repetitions performed and w is the amount of weight. Panel b indicates no significant difference for the motivation of bodybuilders in sham vs. real sessions evaluated through VAS, a continuous single-item fatigue scale ranging from 0 (no fatigue) to 10 (severe fatigue) (p<0.05). Panel c indicates a significant difference in SEI [multiplication of the amount of weight (30% of their 1RM) by the number of successive exercise] for the sham vs. real tDCS session (p<0.05). Panel d shows a significant difference between the RPE in sessions 1 and 2 (p<0.05). Panel e shows a significant difference between HR in the last 12 lifts (the average HR of each two leg extension exercises is plotted in the graph) in sham and real tDCS sessions (p<0.05). Paired *t*-test was used with the *p* value significance level set at 0.05. ns: non-significant.

One-repetition maximum (1RM): With regard to the 1RM used to evaluate the maximum weight lifting performance, the real tDCS vs. sham could improve mean muscular strength score by 4.4% (*p* = 0.002) (Fig 2).

Short-term endurance: The findings revealed that compared to sham, real tDCS could significantly (*p* = 0.004) increase the participants’ mean short-term endurance score (SEI) by 16.9% (Fig 2). This indicates the potential impact of true tDCS vs. sham on muscular endurance.

**Rated** p**erceived** e**xertion:** The analysis revealed a statistically significant difference between the participants’ PE in sessions 1 (sham) and 2 (real) tDCS. The real tDCS vs. sham could decrease RPE mean scores by 14.2% (*p* = 0.007) (Fig 2).

Heart rate: The results of HR recording over the last 12knee extension exercises showed a significant difference between real vs. sham tDCS session and the 6 final *p* values were 0.006, 0.008, 0.03, 0.009, 0.01, and 0.008, respectively, suggesting decreased HR by 4.9% following brain stimulation (Fig 2).

### Motivation

Based on our findings, there was not a statistically significant difference between the participants’ motivation in sham and real tDCS sessions; however, the mean score increased from 6.1 in sham session to 6.5 in real tDCS session (Fig 2).

**Fig 3:**
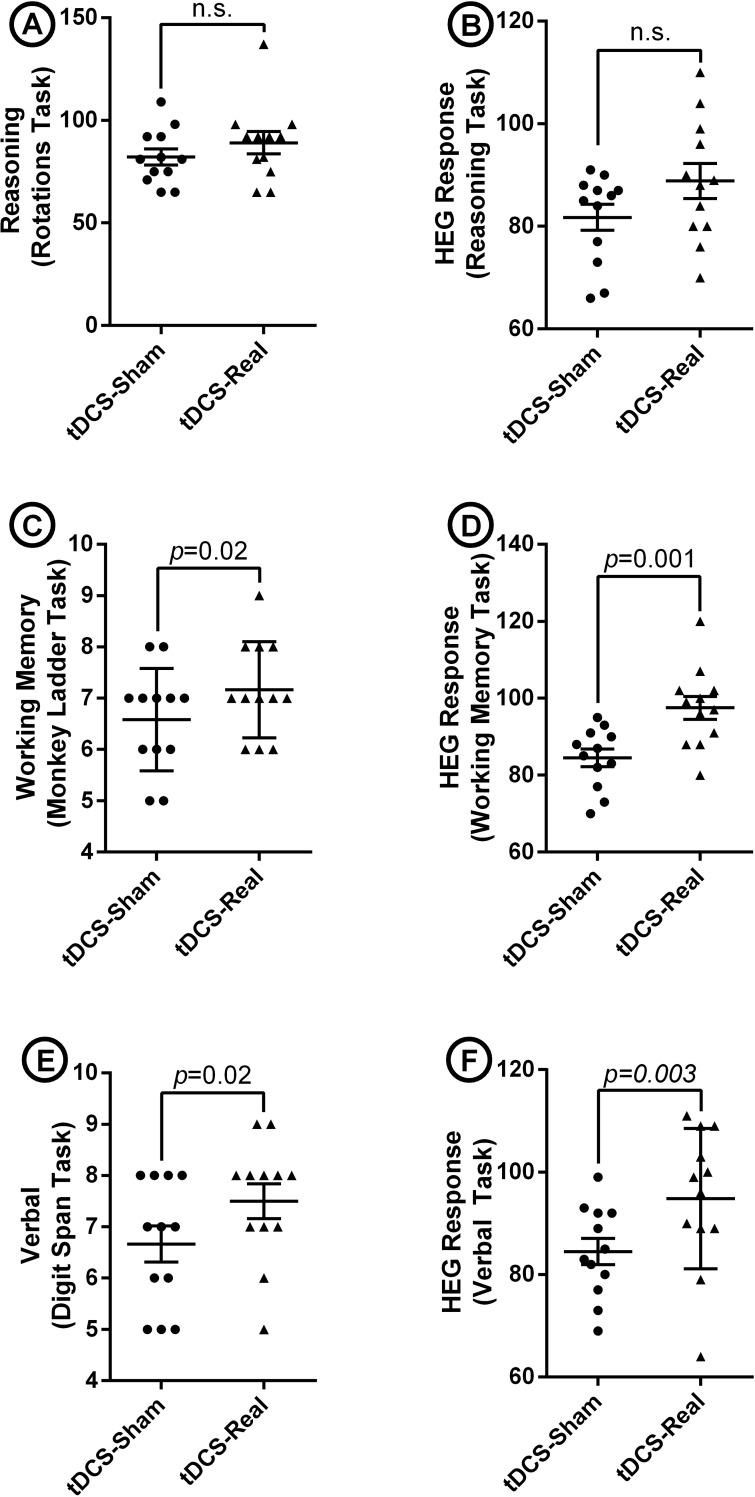
Dot plots showing the performance of bodybuilders in CBS-CP tasks i.e. reasoning, memory, and verbal and the HEG data recorded while taking the tasks over two sessions, 72 hours apart. Panel a shows no significant difference between the performance of the athletes on a reasoning task in sham vs. real tDCS sessions. Panel b indicates no significant difference between the HEG responses upon reasoning task in sham and real sessions. Panel c represents a significant difference in a memory task scores from sham to real tDCS session (p<0.05). Panel d indicates a significant difference between the HEG responses upon a memory task in sessions 1 and 2 (p<0.05). Panel e shows a significant difference between the verbal task scores in sham and real tDCS sessions (p<0.05). Panel f shows a significant difference between the HEG responses upon a verbal task in sham and real sessions (p<0.05). Paired t-test was used with the p value significance level set at 0.05. ns: non-significant.

Cambridge brain sciences-cognitive platform: The results of paired-sample *t*-tests on CBS-CP tasks showed a statically significant difference between the sham and real tDCS sessions in memory (*p* = 0.02) and verbal (*p* = 0.02) tasks. However, with respect to the reasoning task, brain stimulation could not enhance the mean score from sham to real tDCS session (Fig 3).

### Hemoencephalography response

A series of paired-sample *t*-test were used to compare cerebral blood flow in FP1 in the sham and real tDCS sessions. Our findings indicated a statistically significant increase in FP1 hemodynamic response upon memory (*p* = 0.001) and verbal (*p* = 0.003) cognitive tasks (Fig 3). Nevertheless, for the reasoning task, no statistically significant difference was noted in HEG response from session 1 to session 2.

### Surface electromyography (sEMG)

**Fig 4:**
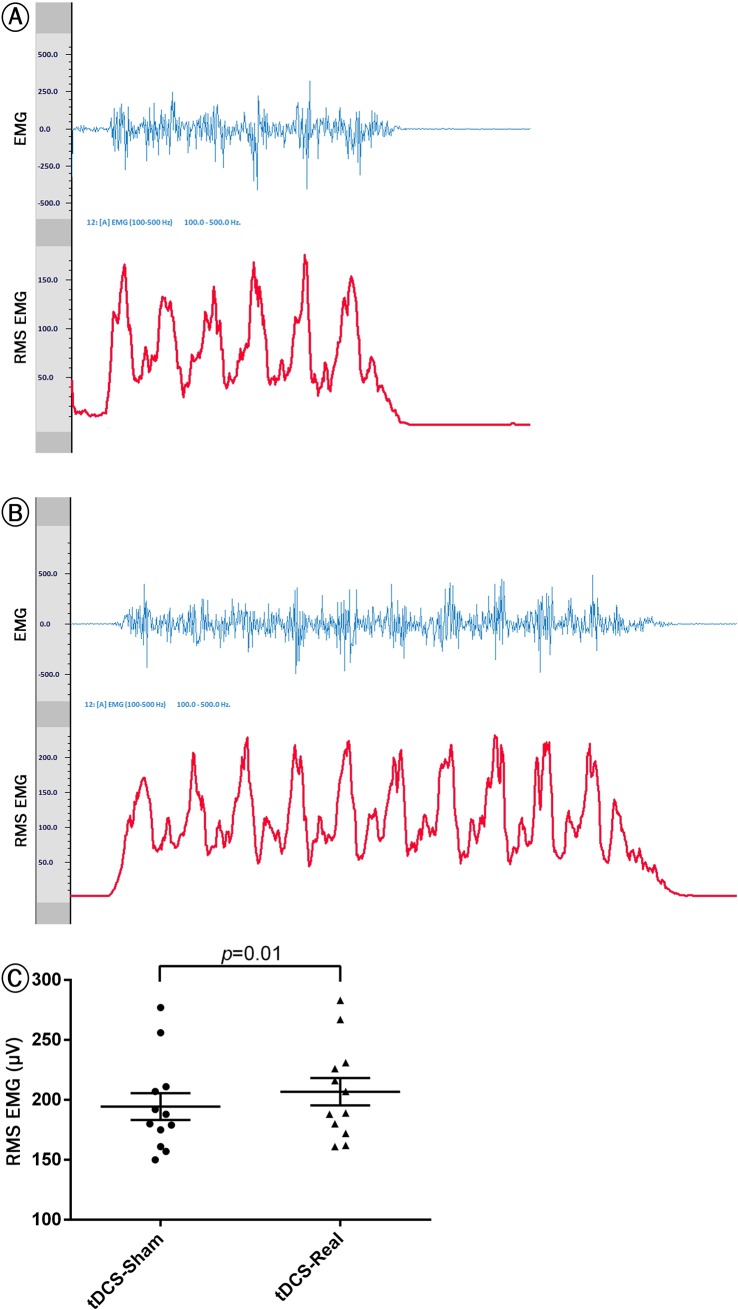
sEMG recording from bodybuilders’ rectus femoris during 1RM exercise. The image represents the ‘raw’ EMG signal of rectus femoris muscle (Blue) and the ‘RMS’ (Root Mean Square) of rectus femoris muscle (Red). Panels a and b represent the gross pattern for muscle contraction including the sEMG amplitudes were calculated (epoch size: 1/8 s, 32 samples per second) after sham and real tDCS, respectively. The peaks correspond to the lifts performed during the 1RM exercise. Comparing the panels a and b, the frequency is shown to get increased after real tDCS. Panel c represents a significant difference in RMS from sham to real tDCS sessions (p<0.05). Paired *t*-test was used with the *p* value significance level set at 0.05. ns: non-significant.

Results of the sEMG indicated that anodal M1 and TC stimulation could significantly increase the sEMG amplitude (*p* = 0.01). Moreover, as shown in Fig 4, the protocol could increase the sEMG frequency during the knee extension lift task (Fig 4).

## Discussion

Over recent years, brain stimulation has become a trend for athletic performance enhancement; however few studies [2, 13, 14, 34] have systematically investigated the issue so far. Nevertheless, most tDCS studies on athletic performance have addressed the issue of athletes’ endurance [9, 14], while the impact of brain stimulation on maximal muscular strength of athletes is yet to be further defined. Taking into account the critical role of brain in exercise performance [35], the present study aimed to determine whether a tDCS-induced cortical excitability of M1 leg area and TC would enhance neural processing and, as a result, improve maximal muscular power, muscular endurance and fatigue, motivation, HR, prefrontal hemodynamic response, and cognitive functions in experienced bodybuilders.

With respect to the athletic performance, ANS activity is shown to be associated with exercise performance [36] and perceived fatigue [37]. Confirming the association between the TC, and ANS [9], specifically HR and blood pressure [36], our findings showed that TC stimulation regulates the athletic performance potentially due to a decreased perception of exertion in relation with the ANS function. Through modulating the ANS, this technique reduced the bodybuilders’ HR by 4.9% during the 12 final moves of the muscular endurance exercise, presumably through increasing and decreasing parasympathetic and sympathetic functions, respectively. Hence, our results proposed the facilitatory effects of tDCS applied over TC associated with ANS activity which has previously been shown to improve cardiac autonomic control during exercise [9, 10, 12, 38].

Indeed, greater vagal modulation is shown to result in regulated cardiovascular autonomic function, which potentially improves athletic performance [39]. Moreover, athletes are shown to have higher vagal modulation than non-athletes [39] and consequently, their heart rate increases more slowly than non-athletes under a specific task [39]. The athletic performance can be enhanced by stimulating specific brain regions related to the autonomic nervous system and increasing vagal modulation. As such, vagal modulation can be assessed by comparing changes in heart rate before and after the tDCS [40]. The vagus nerve fibers are more richly innervated in the atrium than in the ventricle, where energy for the contractions of the heart is provided. This may justify the effect of vagal stimulation, which typically slows down the heart, while the contractibility of cardiac muscles does not reduce much [41]. In fact, a slight reduction in heart rate during an intense exercise may improve cardiac muscles’ contractibility. In our study, the heart rate significantly reduced almost at the end of the physical task.

Moreover, our results indicated that 2mA anodal tDCS for 13 minutes significantly boosted maximal strength and endurance performance of bodybuilders by 4.4% and 16.9%, respectively, compared to sham stimulation. The results of enhanced maximal power (1RM) and endurance (SEI) could justify the impact of anodal tDCS over M1 to improve muscle strength [3, 37], muscle synergy, muscular endurance [7, 8], motor performance of the lower limbs [37], locomotion and balance in patients with Parkinson’s and stroke [42–44]. This has also been extended to more complex tasks such as static [45] and dynamic balance learning though induced excitability [46]. Yet, our results contradict the report showing that the maximum capacity of professional pianists in motor learning is limited [47]. Our findings suggest that the maximal strength of experienced bodybuilders can be enhanced following brain stimulation.

Anodal tDCS is shown to potentially reduce the release of the inhibitory neurotransmitter GABA [48]. This has also been postulated through the evidence from magnetic resonance spectroscopy (MRS) [49, 50] showing that the anodal stimulation inhibits the release of GABA [48, 51]. In the current study, GABA reduction probably improved the functions of cholinergic and glutamatergic neurons [52]. As a result, the excitability of motor neurons increases which may: 1- increase the release of acetylcholine neurotransmission in the synaptic terminal of neuromuscular system, 2-involve more muscular units,3- increase the mean neuronal firing rate [7], and 4-increase 1RM.

Formerly, it was shown that despite improving cycling performance and time to exhaustion, anodal tDCS over M1 did not affect PE and HR factors [13]. Nonetheless, our investigation confirmed positive effects of tDCS on HR and perception of exertion besides enhancing maximal and endurance exercise performance. Our findings were in agreement with the studies which demonstrated the positive effects of anodal stimulation of M1 in decreasing muscle fatigue [3, 7, 8]. Therefore, it may be concluded that simultaneous anodal stimulation of M1 and TC can be an optimized protocol to potentiate the overall performance of athletes considering important athletic factors of muscular power, endurance, fatigue perception, and HR.

The existing evidence support the fact that the temporal region retains a defining role in the functional regulation of autonomic nervous system [10]. According to a study, when people have a good feeling, like when a mother sees her child's photo, their left insular cortex in activated [53]. In the current study, in addition to the motor cortex, the temporal cortex was concurrently stimulated. This has potentially resulted in a reduced perceived exertion (rated through RPE) and heart rate, supporting the effect of anodal tDCS on autonomic functions.

In our study, the enrolled athletes rated their perceived intensity of a physical exercise using the RPE. The RPE results are used to determine the maximum exertion in a physical exercise. Studies have shown that the RPE scale is well correlated with serum as well as muscle lactic acid, which are biochemical markers for muscle fatigue [28]. Therefore, an open question to address in future research is to investigate whether reduced RPE is proportionately linked to altered levels of serum and muscle lactic acid.

Being involved in emotional awareness and recognizing emotional stimuli, TC was stimulated in order to examine its effect on the bodybuilders’ motivation. In agreement with the finding of the reports showing the positive effects of tDCS over the right motor cortex of healthy subjects in improving motivation [7], our study similarly found that the motivation mean score increased. However, statistical analysis did not reveal any significant difference between sham and real tDCS. This might be preliminarily due to the focus of the tDCS since we have not placed the anodal stimulation on the specific areas (frontopolar brain regions) [54] which are potentially linked to the motivational capacity of the participants undergoing the training exercise. Although the anodal stimulation of T3 may result in positive feelings [9], it could not significantly improve the bodybuilders’ level of motivation in our study.

With respect to cognitive functions, according to Furley *et al*. in addition to muscular performance, the cognitive functions such as working memory and reasoning, are among potentially defining factors in athletic performance mainly at professional level. An enhanced focus and working memory function can reduce executive lapses and improve tactical decisions in sport competitions [55]. The cross-link between cognitive capacity and athletic performance following transcranial electrical stimulation need to be defined not only to show the safety of applied stimulation but also the possible benefits on athletic performance.

To assess the cognitive aptitude of examinees in relation to muscular performance following cortical electrical stimulation, we chose to employ the Cambridge Brain Science-Cognitive Platform (CBS-CP). This tool is among the mostly used and validated media-rich computer platforms with an ongoing normative database comprising over 40000 subject entries worldwide [32]. Since the peak effect of single session tDCS remains for almost one hour [27].we faced time-constraint and had to limit cognitive assessments to maximum 3 tasks. As such, one task from each domain within the CBS-CP (i.e. Memory, verbal and reasoning) was selected and administered. More comprehensive cognitive assessments would better be assessed in future research works of similar context where time-constraint is not an issue.

In the current study, the motor cortex stimulation resulted in the enhancement of working memory, which could also affect long-term memory. Proji *et al*. [56] showed that anodal tDCS over the primary motor cortex (M1) would potentiate synaptic plasticity through modulating NMDA receptors and ultimately result in the enhancement of long-term memory.

One way to measure neuronal activity is to record cortical hemodynamic changes [57]. The hemodynamic changes in the left frontopolar cortical region (FP1) can be measured using hemoencephalography (HEG) response. Based on the existing evidence, a higher HEG response at prefrontal cortical regions corresponds to proper cognitive capacity [57]. In our study, there was probably a direct relationship between the cognitive function improvement (memory and verbal) and increased HGE response following anodal tDCS.

Our study was consistent with that of Toomim *et al*. conducted on people with attention deficit showing that people with increased HEG response in FP1 scored higher in the TOVA test (Test Of Variables of Attention) which assesses several cognitive and behavioral domains including attention, reaction time and impulsivity [58]. In our study, the cognitive test scores (Digit Span and Monkey Ladder Tests) were significantly improved proportionately with the gain in HEG response. These two tests are categorized as visuospatial and verbal working memory testing tools [32]. A key large-scale brain network involved in visuospatial scanning and working memory is located within the prefrontal cortex where the HGE response significantly increased in our investigation [32]. Since in addition to the muscular strength, cognitive functions are also effective factors in athletic performance, we investigated three different cognitive tasks taking into consideration the research limitations. Our intervention was shown to improve some cognitive domains and this was reflected in frontopolar hemodynamic changes.

Considering the cognitive functions, anodal tDCS over the M1 leg area and TC was not only safe in terms of hampering cognitive functions but also effective in improving mean scores in some domains memory and verbal ability tasks. However, no significant effect was noted regarding the reasoning task. Outperformance on ‘Digit Span task’ (verbal) could be justified by repetition in the task associated with superior longitudinal fasciculus and arcuate fasciculus modulated through T3 stimulation. The results on verbal task could be compared with a study [59] indicating the positive effects of tDCS over M1 in word-retrieval, a verbal task. However, the results are in contrast to earlier researches which showed that anodal tDCS over M1 had no significant effect on working memory in healthy individuals [60] and patients with Parkinson’s disease [61].

We preliminary hypothesized that the safety of this intervention would need to be warranted by no potential impairment in cognitive capacity of the participants who underwent this process, surprisingly, it was found that not only they did not have any decline in cognitive function, but in some specific domains it showed to have outperformance in terms of memory and verbal capacity. This could potentially be justified through stimulated networks which are practically implicated in cognitive functions including memory and verbal ability. Moreover, tDCS induced excitability resulted in increase in prefrontal hemodynamic response upon memory and verbal cognitive tasks as suggested by previous studies showing an increased blood-oxygen level due to anodal brain stimulation [6, 62] which is probably caused by cortical excitability [5].

Moreover, anodal M1/TC stimulation could increase sEMG amplitude (RMS) in line with the reported effectiveness of anodal motor stimulation on biceps brachii muscle activation and increase in elbow flexor muscle activity [63]. However, the results cannot be generalized to all age groups since it is already revealed that tDCS over the motor cortex in old adults exerted no effects on their elbow flexion muscle strength and sEMG amplitude [64]

Moreover, the Type II fast muscle fibers are often innervated through high-threshold neurons. This type of muscle fibers is often closer to the surface and their contraction variations can be well traced with the sEMG [8]. As a result, a small change in using motor units can be traced with the sEMG [65]. In the current study, a significant increase in RMS was observed following tDCS reflecting empowered motor units during an isotonic task. However, further studies are required to determine a more accurate mechanism for muscular activities following tDCS.

The current study was subject to some shortcomings including a relatively small sample size, lack of further objective assessments techniques to label fatigue such as magnetic resonance spectroscopy of muscles for lactic acid level, lack of further dose-response examinations in tDCS including varied protocols based on timing and amplitude. In addition, brain mapping upon bodybuilders’ task performance through quantitative electroencephalography (qEEG) would be of great help in future research.

Although this study indicated the efficiency of simultaneous anodal stimulation of M1 leg area and TC in enhancing the performance of bodybuilders in terms of muscular strength, endurance, HR, fatigue, prefrontal hemodynamics, sEMG amplitude, and cognitive ability, further investigations should attempt to define optimized protocols to be used in real practice of bodybuilders.

Furthermore, due to the important role of cerebellum in movement and muscular coordination and strength as well as its close relationship with motor cortex, the question about application of cerebellar tDCS for bodybuilders’ performance can be sought is future research.

With a larger sample size, another issue to tackle is to examine whether there is a correlation between athletic performance in bodybuilders (i.e. 1RM) and cognitive profile. Some cognitive domains including emotion, drive, motivation and attention may potentially be linked to optimized performance in athletic field [66, 67]. Though, the hypothesis would need to be systematically addressed in future well-designed research.

Finding from this research is expected to add to the emerging body of evidence toward incorporating applied neuroscience insights in to sports. Further systematic research on similar topics need to gain momentum to bring such novel insights in to real life applications.

## Conclusion

Taken together, our present report suggests that the integration of anodal M1 leg area and TC tDCS may assist bodybuilders to improve their overall performance. This study may pave the path towards designing brain stimulation protocols to enhance strength and subsequently the muscle mass in bodybuilding which is known as a basic competency in many sports. Additionally, since sustainable training may hardly affect the autonomic nervous system tone, auxiliary methods such as tDCS to assist athletes with decreased fatigue perception could be worthwhile.

The present results may appeal to the interest of athletes, coaches and policy makers to help improve athletic performance.

## Supporting information

S1 Table Raw data. (XLSX)

S2 Table CONSORT checklist. (DOC)
