# Supplementary material for: Transcranial direct current stimulation to enhance athletic performance outcome in experienced bodybuilders
Source: PLoS One. 2019 Aug 1;14(8):e0220363. doi: 10.1371/journal.pone.0220363 (PMC6675286; doi:10.1371/journal.pone.0220363)
Supplement: S2 Table — (DOC) [file pone.0220363.s002.doc]

**
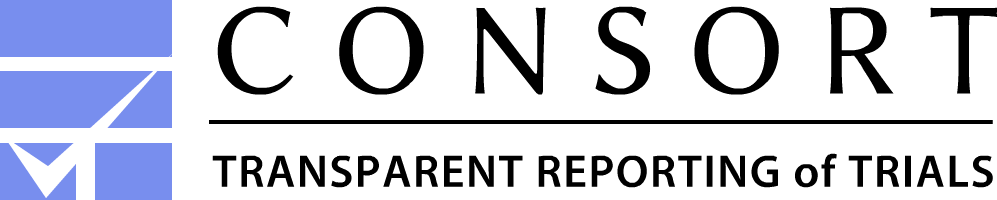
**

**CONSORT 2010 Flow Diagram**

**Allocation**

**Analysis**

**Follow-Up**

**Enrollment**

Assessed for eligibility (n=26)

Excluded (n=14)

♦  Not meeting inclusion criteria (n=9)

♦  Declined to participate (n=3)

♦  Other reasons (n=2)

Analysed (n=12)
♦ Excluded from analysis (n= 0)

Lost to follow-up (n=0)

Discontinued intervention (n=0)

Allocated to Sham- tDCS condition (n= 12)

♦ Received allocated intervention (n= 12)

♦ Did not receive allocated intervention (n= 0)

Lost to follow-up (n=0)

Discontinued intervention (n=0)

Allocated to Real-tDCS condiiton (n=12)

♦ Received allocated intervention (n=12)

♦ Did not receive allocated intervention (n=0)

Analysed (n=12)
♦ Excluded from analysis (n=0)

Randomized (n= 12)
